# High-Throughput System for the Early Quantification of Major Architectural Traits in Olive Breeding Trials Using UAV Images and OBIA Techniques

**DOI:** 10.3389/fpls.2019.01472

**Published:** 2019-11-18

**Authors:** Ana I. de Castro, Pilar Rallo, María Paz Suárez, Jorge Torres-Sánchez, Laura Casanova, Francisco M. Jiménez-Brenes, Ana Morales-Sillero, María Rocío Jiménez, Francisca López-Granados

**Affiliations:** ^1^Department of Crop Protection, Institute for Sustainable Agriculture (IAS), Spanish National Research Council (CSIC), Córdoba, Spain; ^2^Departamento de Ciencias Agroforestales, ETSIA, Universidad de Sevilla, Sevilla, Spain

**Keywords:** remote sensing, unmanned aerial vehicle, table olive, breeding program, training system, tree crown area and volume, point cloud

## Abstract

The need for the olive farm modernization have encouraged the research of more efficient crop management strategies through cross-breeding programs to release new olive cultivars more suitable for mechanization and use in intensive orchards, with high quality production and resistance to biotic and abiotic stresses. The advancement of breeding programs are hampered by the lack of efficient phenotyping methods to quickly and accurately acquire crop traits such as morphological attributes (tree vigor and vegetative growth habits), which are key to identify desirable genotypes as early as possible. In this context, an UAV-based high-throughput system for olive breeding program applications was developed to extract tree traits in large-scale phenotyping studies under field conditions. The system consisted of UAV-flight configurations, in terms of flight altitude and image overlaps, and a novel, automatic, and accurate object-based image analysis (OBIA) algorithm based on point clouds, which was evaluated in two experimental trials in the framework of a table olive breeding program, with the aim to determine the earliest date for suitable quantifying of tree architectural traits. Two training systems (intensive and hedgerow) were evaluated at two very early stages of tree growth: 15 and 27 months after planting. Digital Terrain Models (DTMs) were automatically and accurately generated by the algorithm as well as every olive tree identified, independently of the training system and tree age. The architectural traits, specially tree height and crown area, were estimated with high accuracy in the second flight campaign, i.e. 27 months after planting. Differences in the quality of 3D crown reconstruction were found for the growth patterns derived from each training system. These key phenotyping traits could be used in several olive breeding programs, as well as to address some agronomical goals. In addition, this system is cost and time optimized, so that requested architectural traits could be provided in the same day as UAV flights. This high-throughput system may solve the actual bottleneck of plant phenotyping of “linking genotype and phenotype,” considered a major challenge for crop research in the 21st century, and bring forward the crucial time of decision making for breeders.

## Introduction

The olive tree (*Olea europaea* L.) area amounts to more than 10 million hectares world-wide, with over 97% of this being concentrated in the Mediterranean Basin (FAOSAT, 2017; IOC, 2017). The olive industry plays a key economic role in this area, since it accounts for 96% of the world’s olive production, i.e. 18.5 million tons approximately. Spain leads the world ranking both in production and surface area, followed by Greece, Italy, and Turkey (FAOSAT, 2017). In addition, Mediterranean countries are the largest consumers of olive oil with a quota about two-thirds of world consumption (IOC, 2017). Besides being one of the most important agro-food chains in the Mediterranean Basin, olive growing constitutes a key element of rural society as a significant source of income and employment for rural populations (Stilliano et al., 2016). Furthermore, olives are expanding to many regions outside the Mediterranean Basin such as the United States, Australia, China, and South Africa as well as other sub-tropical and warm temperate areas, making the olive tree the most extensively cultivated fruit crop in the world (FAOSAT, 2017). Besides, olive products are very appreciated not only as healthy food, but also in medical and cosmetic use (Fabbri et al., 2009).

The need for the modernization of olive farms in producing countries and its diffusion outside traditional areas of growth have led to farm investments to improve the productive framework through more efficient crop management strategies, such as irrigation, pruning and harvesting mechanization, and new training systems (e.g. super-high-density hedgerow). These new growing techniques are encouraging the development of cross-breeding programs to release new olive cultivars more suitable for mechanization and use in intensive orchards, with high quality production and resistance to biotic and abiotic stresses (Fabbri et al., 2009; Stilliano et al., 2016; Rallo et al., 2018). Plant breeding programs have benefited from recent advances in genomics and biotechnology by improving genotyping efficiency (Rugini et al., 2016), whereas the lack of efficient phenotyping methods still represents an important bottleneck in these programs (White et al., 2012). Traditional methods to collect phenotypic data (i.e. observable morphological traits related to growth, development, and physiology) rely on manual or visual sampling, which is time-consuming and laborious (Madec et al., 2017; Yang et al., 2017). Improving the acquisition of crop traits such as morphological attributes, flowering time, and yield has therefore become the main challenge limiting designing and predicting outcomes in breeding programs (Zaman-Allah et al., 2015). This aspect is particularly crucial for olive breeding due to the large genetic variability commonly obtained in seedling progenies (Rallo et al., 2018), coupled with the great complexity of collecting data on common large olive plots, which requires major logistical considerations (Araus and Cairns, 2014).

To overcome the challenge of automated and fast collection of phenotypic crop data, high-throughput phenotyping platforms have become crucial due to their ability to rapidly phenotype large numbers of plots and field trials at a fraction of the cost, time, and labor of traditional techniques (White et al., 2012; Zaman-Allah et al., 2015; Yang et al., 2017). Among the high-throughput phenotyping platforms for non-destructive plant data collection under field conditions such as autonomous ground vehicles (Shafiekhani et al., 2017; Virlet et al., 2017), tractor-mounted (Montes et al., 2007), pushed platforms (Bai et al., 2016), or cable-driven (Newman et al., 2018); unmanned aerial vehicles (UAVs) have been highlighted due to their capacity to generate field scale information using a wide range of sensors and operating on demand at critical moments and at low flight altitude, thus meeting the critical requirements of the spatial, spectral, and temporal resolutions of breeding programs (Shi et al., 2016; Tattaris et al., 2016; Yang et al., 2017; Ostos et al., 2018; Torres-Sánchez et al., 2018a). Nevertheless, little information exists on the use of UAVs for olive breeding. In this regard, Díaz-Varela et al. (2015) used a camera on board a UAV platform to estimate tree height and crown diameter in both discontinuous and continuous canopy systems of olive orchards. However, early phenotyping of olive trees (i.e., phenotyping in the first few years after planting) using UAVs has not been addressed. The genotype evaluation in olive cross-breeding programs usually follows a multi-step protocol that includes the initial evaluation of seedlings and their successive clonally propagated selections in field trials (Rallo et al., 2018). Each of these field stages (seedlings, pre-selections, advance selections, comparative trials) involves a high cost of maintaining a large number of trees over the years required for the evaluation of the target traits according to the breeding goals. Tree vigor and other architectural traits are relevant parameters to be evaluated in any of these breeding stages, since early vigor is known to be related to the juvenile period length in seedlings (De la Rosa et al., 2006; Rallo et al., 2008), and vegetative growth habits are key to evaluate the suitability of selected genotypes to be cultivated under different planting systems, such as superhigh density hedges (Hammamia et al., 2012; Rosati et al., 2013). Therefore, the ability to quantify these traits through cost-efficient methods in young trees would allow the identification of desirable genotypes as early as possible, thus saving time, labor, and money (Rallo et al., 2018). In addition, the knowledge of tree geometry can be used as a valuable tool to design site-specific management strategies (De Castro et al., 2018a).

Geometric traits can be estimated from 3D point clouds or Digital Surface Models (DSMs) based on UAV-imagery due to the ability of UAVs to fly at low altitudes with high image overlap (Torres-Sánchez et al., 2015; Yang et al., 2017; De Castro et al., 2018b). In the context of woody crops, these 3D models offer the rapid and accurate assessment of growth traits in poplar (Peña et al., 2018), vineyard (Matese et al., 2017; De Castro et al., 2018a), almond (Torres-Sánchez et al., 2018b), lychee (Johansen et al., 2018), and olive (Díaz-Varela et al., 2015; Torres-Sánchez et al., 2015; Jiménez-Brenes et al., 2017). Among these approaches, 3D point clouds have been highlighted for improving 3D reconstruction as they provide more height information (Z-value) at each coordinate (X,Y), while DSMs are defined as 2.5D datasets as they have only one height value at each 2D coordinate (Monserrat and Crosetto, 2008; Torres-Sánchez et al., 2018b). However, the large amount of detailed crop data embedded in the UAV-based 3D point clouds information requires the development and implementation of robust image analyses. In this regard, object-based image analysis (OBIA) techniques have reached high levels of automation and adaptability to high-data images. Furthermore, OBIA overcomes the limitations of pixel-based methods by segmenting images into groups of adjacent pixels with homogenous spectral values, called “objects”, which are used as basic elements of the classification analysis where spectral, topological, and contextual information are combined, thus providing successful automatic classifications in complicated agricultural scenarios (Blaschke et al., 2014; Peña et al., 2015; López-Granados et al., 2016; De Castro et al., 2018b).

As per the above discussion, a UAV-based high-throughput system was developed and tested in experimental trials within an olive breeding program with the aim to quantify plant architectural traits of very young olive trees. To achieve this goal, a full protocol to collect the UAV images and create 3D point clouds was described, and a novel and customizable 3D point cloud-based OBIA was developed to characterize the 3D structure of the young plants, measured by tree height, crown area, and volume, in the first two years after planting, without any user intervention. In addition, the potential applications of these estimated olive plant traits for olive breeding programs were discussed.

## Materials and Methods

### Study Fields

**Figure 1:**
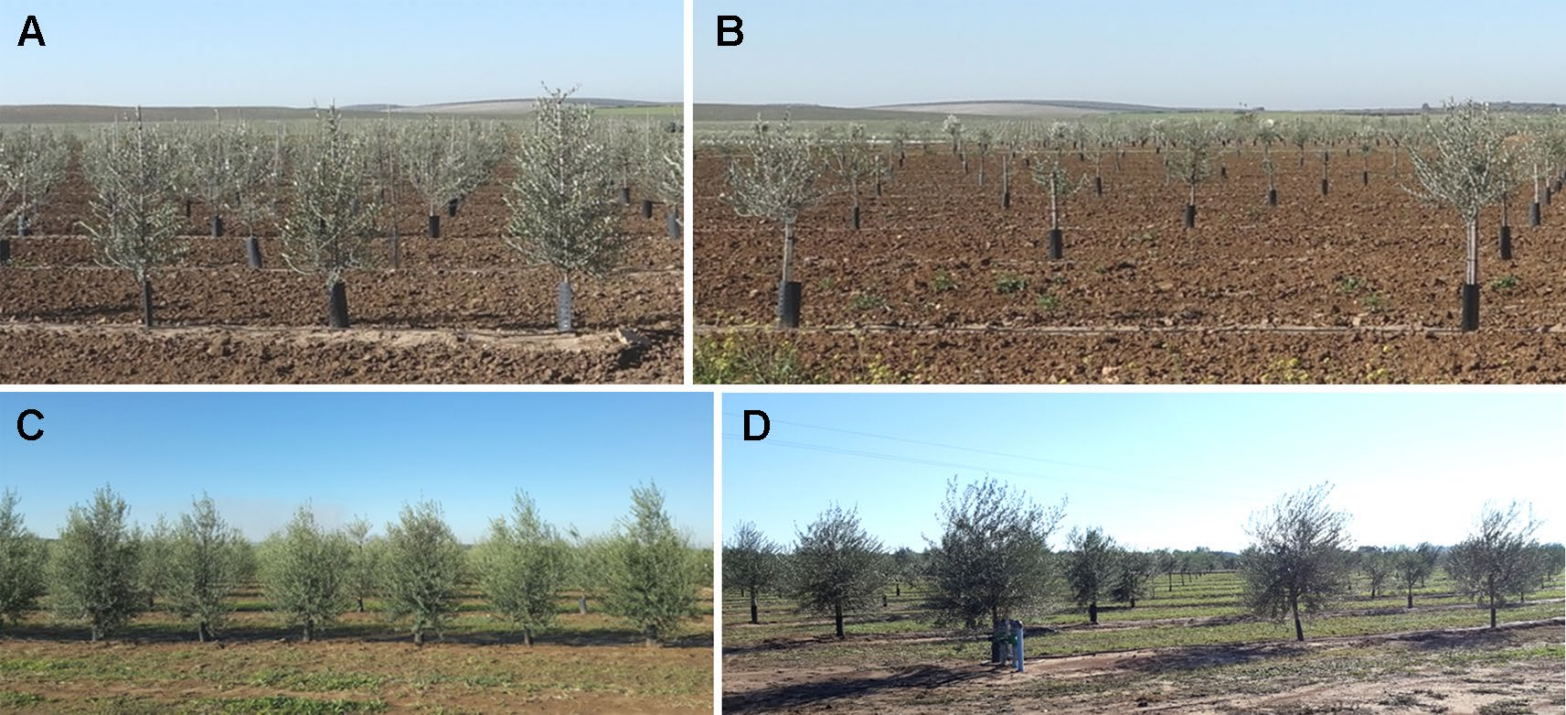
General view of the olive field trial studied: **(A)** hedgerow trial and **(B)** intensive trial in 2017; and **(C)** hedgerow trial; and **(D)** intensive trial in 2018.

The experiment was carried out in two field trials located in Morón de la Frontera, Sevilla (Southern Spain). Both fields were planted in October 2015 in the framework of the University of Sevilla table olive breeding program, which were drip-irrigated, with flat ground and an approximate surface area of 1.20 ha each. The two trials were selected to account for differences in training systems: the intensive discontinuous canopy (intensive trial) and the super high density continuous hedgerow (hedgerow trial). The first trial (intensive trial) consisted of trees planted at a 7 × 5-m spacing (286 trees/ha) in a north–south orientation as single trunk open vase forming a discontinuous canopy of scattered trees (**Figures 1B, D**). Twenty-six olive genotypes (10 trees per genotype) were included in the intensive trial in a randomized design with two trees per elementary plot and five repetitions. In the second trial (hedgerow orchard), olive trees were planted in a 1.75 × 5 m pattern (1143 trees/ha) and trained to a central leader system, designed to form a continuous canopy later in crop development (**Figures 1A, C**). The hedgerow trial comprised of 14 olive genotypes arranged in a randomized design with rows of 20 trees per elementary plot and three repetitions (60 trees per genotype). The experiment was carried out at two different early stages of tree development: 15 months after planting, i.e., when the plants completed their first growth cycle in the field; and 27 months after planting, after 2 years in the field, which corresponded with each flight date. No pruning was performed during the experimental period to allow the genotypes following their own growth habit.

### UAV-Based Phenotyping Platform

**Figure 2:**
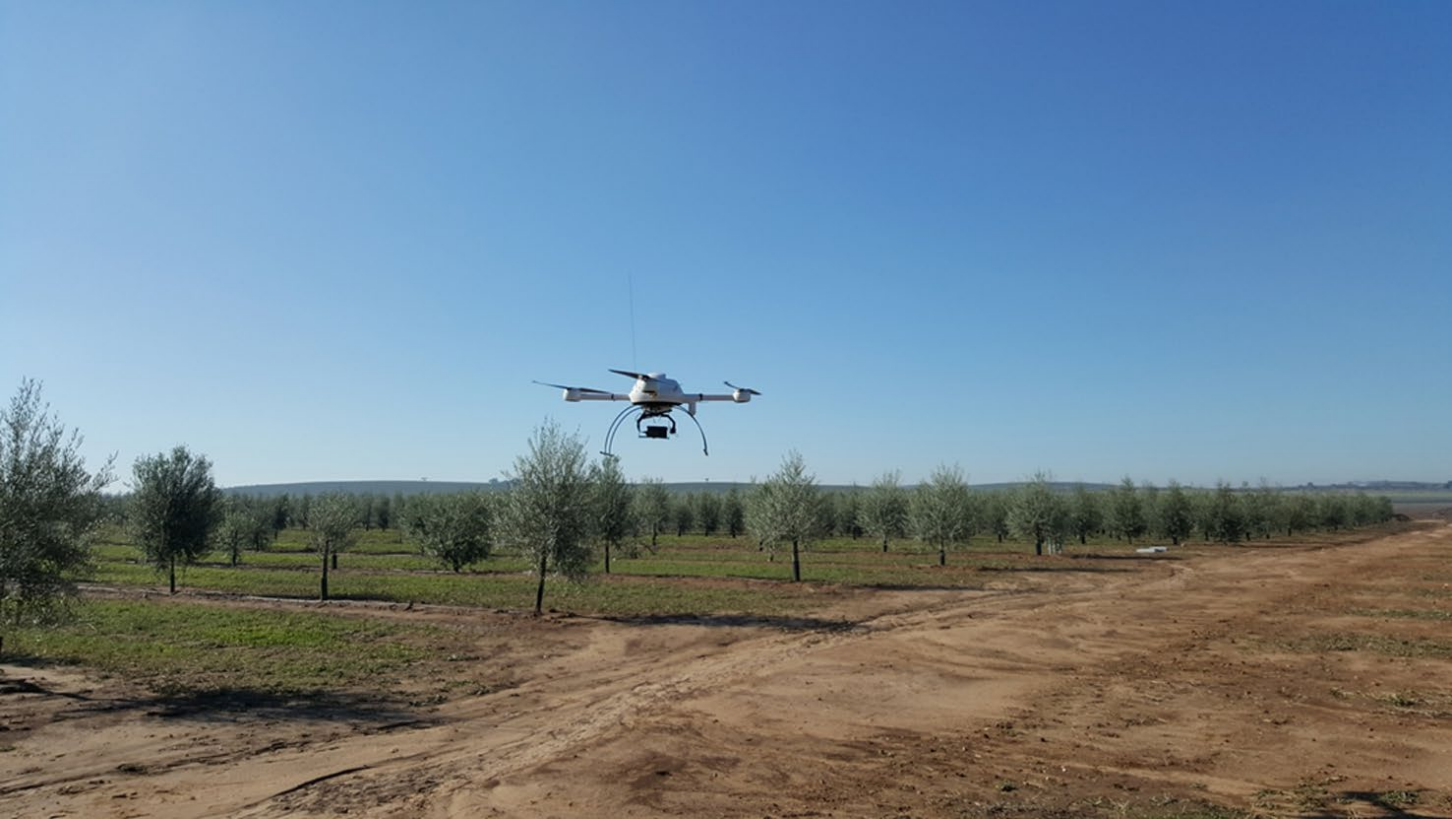
The MD4-1000 UAV flying over the intensive trial in the second studied date (January 2018).

The remote images were acquired at midday on 16th January 2017 and 10th January 2018 with a low-cost commercial off-the-shelf camera, model Sony ILCE-6000 (Sony Corporation, Tokyo, Japan) mounted in a quadcopter model MD4-1000 (microdrones GmbH, Siegen, Germany), which was modified and calibrated to capture information in both NIR and visible light (green and red) by removing the internal NIR filter commonly present in the visible cameras and adding a 49-mm filter ring to the front nose of the lens, all done by the company Mosaicmill (Mosaicmill Oy, Vantaa, Finlandia) (**Figure 2**). This model has a 23.5 × 15.6 mm APS-C CMOS sensor, capable of acquiring 24 megapixel (6,000 × 4,000 pixels) spatial resolution images with 8-bit radiometric resolution (for each channel), and is equipped with a 20 mm fixed lens. The flights were carried out at the same time as the on-ground data were taken to ensure the same meteorological conditions, which consisted of sunny days with calm winds. Moreover, similar weather conditions were reported between flight campaigns.

The UAV can either be manually operated by radio control (1,000 m control range) or execute user-defined flight routes autonomously by using its Global Navigation Satellite System (GNSS) receiver and its waypoint navigation system. The UAV is battery powered and can load any sensor weighing up to 1.25 kg. The camera was mounted in the UAV facing downward for nadir capture, and the UAV routes were designed to take images at 50 m flight altitude, resulting in a spatial resolution of 1 cm pixel size, and with forward and side overlaps of 93% and 60%, respectively, which are large enough to achieve the 3D reconstruction of olive orchards, according to previous research (Torres-Sánchez et al., 2015; Torres-Sánchez et al., 2018a). Every yearly campaign consisted on a unique 15-min flight for both field trials that covered a surface of 5 ha. The flight operations fulfilled the list of requirements established by the Spanish National Agency of Aerial Security including the pilot license, safety regulations, and limited flight distance (AESA, 2017).

### Point Cloud Generation

**Figure 3:**
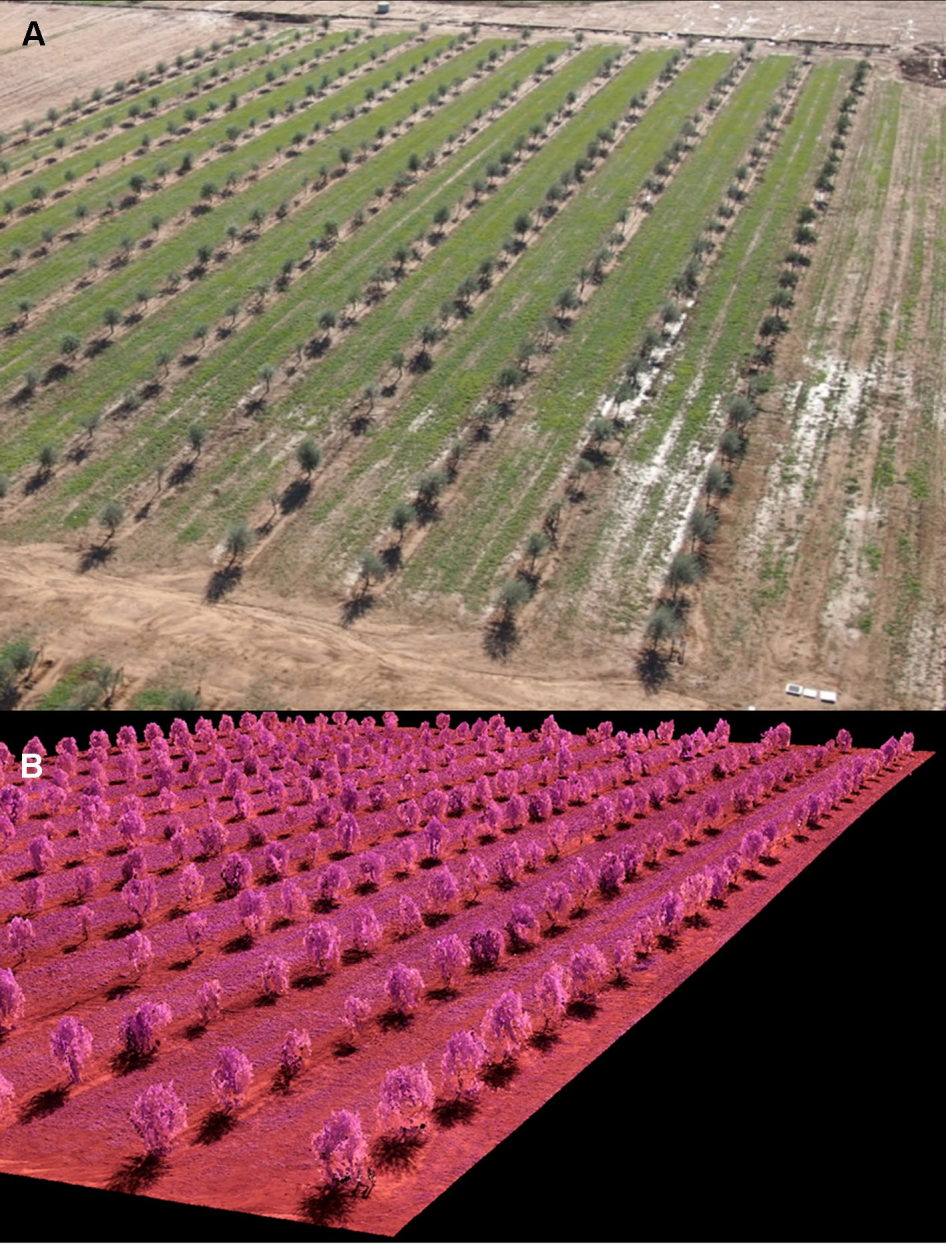
**(A)** General View of the Intensive Field Trial-2018; and **(B)** Partial View of the Corresponding 3D Point Cloud Produced by the Photogrammetric Processing of the Remote Images Taken With the UAV Platform.

A 3D point cloud was generated by using the Agisoft PhotoScan Professional Edition software (Agisoft LLC, St. Petersburg, Russia) version 1.4.4 build 6848. The process was fully automatic, with the exception of the manual localization of six ground control points in the corners and in the center of each field trial with a Trimble R4 (Trimble, Sunnyvale, CA, USA) to georeference the 3D point cloud. The GPS worked in the Real Time Kinematic (RTK) model linked to a reference station of the GNSS RAP network at the Institute for Statistics and Cartography of Andalusia (IECA), Spain. This GNSS-RTK system provided real time-corrections that resulted in an accuracy of 0.02 m in planimetry and 0.03 m in altimetry. The whole automatic process involved two main stages: 1) aligning images, and 2) building field geometry. First, the camera position for each image and common points in the overlapping images were located and matched, which facilitated the fitting of camera calibration parameters. Next, the point cloud was built based on the estimated camera positions and the images themselves by applying the Structure from Motion (SfM) technique (**Figure 3**). Thus, every point consisted of x, y, and z coordinates, where z represents the altitude, i.e., the height above sea level. The point cloud files were saved in the “.las” format, a common public file format that allows the exchange of 3D point cloud data. More details about the software processing parameters are given in De Castro et al. (2018a).

### OBIA Algorithm

**Figure 4:**
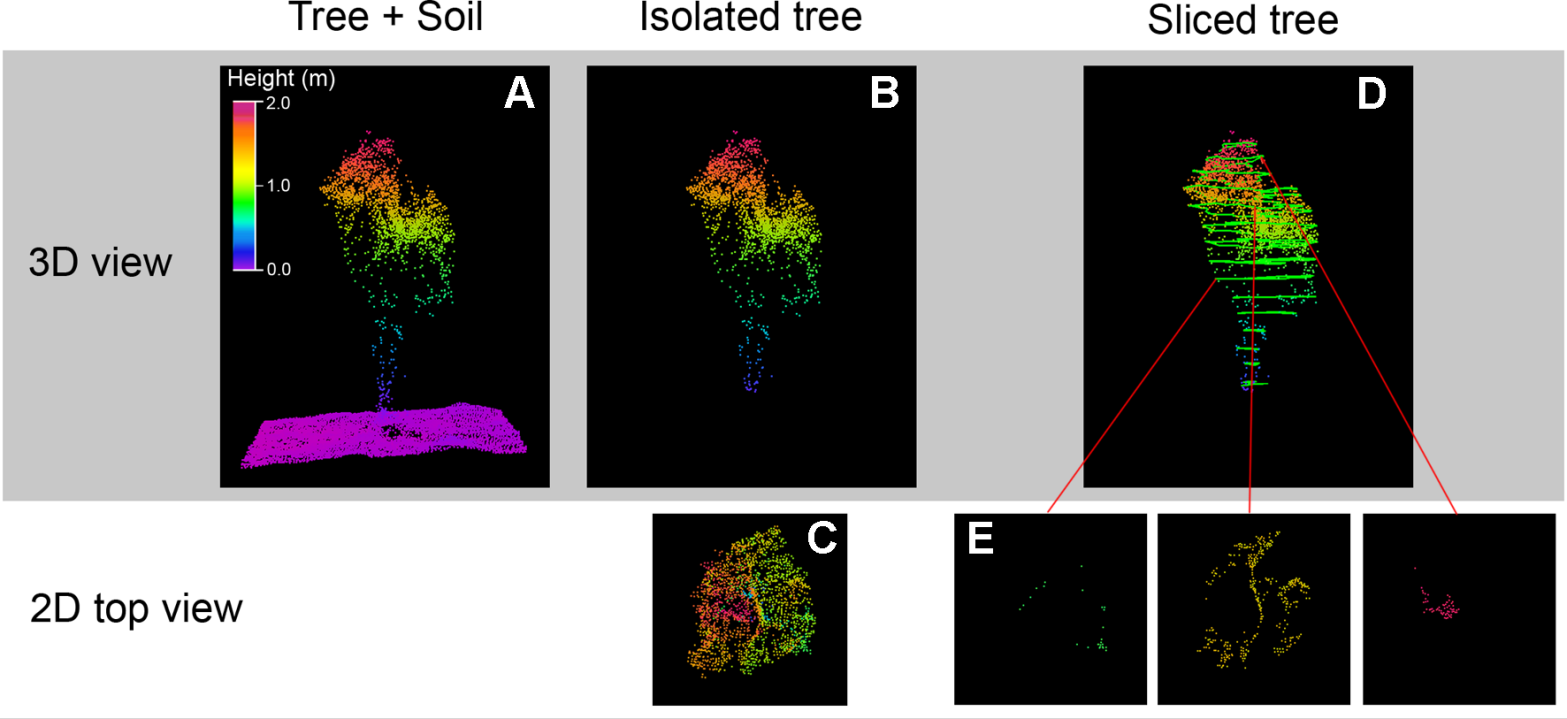
Graphical examples of the Object Based Image Analysis (OBIA) procedure outputs for identification and characterization of the olive seedling: **(A)** 3D point cloud for a square of 2 m side size, DTM is represented in pink color; **(B)** tree point cloud; **(C)** 2D representation, following the x,y axes, of the tree point cloud; **(D)** sliced tree point cloud along Z axis; **(E)** points belonging to the point cloud included in the selected slice portion.

The OBIA algorithm for the identification and characterization of the olive seedling was developed with Cognition Network programming language in the eCognition Developer 9.3 software (Trimble GeoSpatial, Munich, Germany). The algorithm is fully automatic, it therefore requires no user intervention, and was composed of a sequence of phases (**Figure 4**), using only the 3D point cloud as input, as follows:

1. *Digital Terrain Model (DTM) generation*: A chessboard segmentation algorithm was used to segment the point cloud in squares of 2 m side size based on the studied olive tree dimension and the planting patterns (**Figure 4A**). Each square was then assigned a height value corresponding to the average of 15% of the lowest height points to create the DTM layer (**Figure 4A**), i.e., a graphical representation of the terrain height without any objects like plants and buildings, as based on previous studies (Torres-Sánchez et al., 2018b).
2. *Tree point cloud creation*: First, the height above the terrain of every point composing the cloud was obtained based on the DTM. Next, the 0.3 m value was used as the suitable threshold to accurately identify tree points and therefore create the tree point cloud (**Figure 4B**). This threshold was based on the tree size in the stage studied and the lack of cover crops. The height threshold is an easily implemented and accurate tool used for olive detection, either from the UAV photogrammetric point cloud (Fernández et al., 2016) or terrestrial laser scanner point cloud (Escolà et al., 2017).
3. *Tree crown delineation*: After tree point identification, a grid of 0.1 m size was overlaid on the terrain and every projected square with the presence of a tree point was classified as tree, which were then merged to compound each individual tree crown. This parameter was fixed to 0.1 m to be well suited for tree reconstruction using UAV imagery (Torres-Sánchez et al., 2018b).
4. *Point cloud slicing*: Once the tree point cloud was divided into 2D-squares following the x,y axes, the tree point cloud was then segmented into slices from bottom to top along the Z axis according to intervals of 0.1 m (**Figure 4D**) resulting in 3D-grids (voxels) with 0.1 m side. Therefore, the point cloud was included into a tridimensional regular grid composed by small volumetric units (voxels) to be processed. Next, a new image layer called “Voxels” with a resolution of 0.1 m, similar to the voxel size, was created at the ground level, where each pixel stored the number of voxels above (i.e. voxels with the same x,y coordinates at different heights) containing points of the olive crown. The voxel size was set at 0.1 m according to Phattaralerphong and Sinoquet (2005), who reported that the optimal voxel sizes for crown volume estimates ranged from 10 to 40 cm. The size of the voxel has previously been related to the accuracy of the crown volume estimate (Park et al., 2010; Li et al., 2013; Zang et al., 2017), the larger the voxel size, the greater the estimation accuracy. However, oversized voxels lead to the creation of few voxels resulting in statistically insignificant descriptions of canopy features. Thus, taking into account the small size of the olive trees, 0.1 m was selected as the optimal voxel size.
  The voxel-based methodology is considered one of the more advanced techniques for accurately reproduced the tree (Hosoi and Omasa, 2006), where the voxel is the smallest information unit element of a three-dimensional matrix. This methodology allows process the coordinates of each voxel, analyze 3D-models as digital images and consider points measured from successive shots as a single voxel without oversampling (Fernández-Sarría et al., 2013), making voxel-based methodology one of the most useful methods in point cloud analysis. It has been successfully used in tree point cloud analysis generated by LiDAR (Hosoi and Omasa, 2006; Fernández-Sarría et al., 2013; Underwood et al., 2016) and photogrammetric techniques (Gatziolis et al., 2015; Dandois et al., 2017; Torres-Sánchez et al., 2018b).
  Due to the difficulty in obtaining information inside the tree crown of the UAV-photogrammetric approach, the squares surrounded by the crown limit were classified as tree crown, and those voxels taken into account in the process.
5. *Olive tree characterization*: For every olive tree, the volume occupied by the crown was automatically quantified in each pixel of the “Voxels layer” by multiplying the number stored, i.e. voxels containing olive crown points, and the voxel volume (0.1 × 0.1 × 0.1 m^3^). Similarly, Underwood et al. (2016) calculated the crown volume in almond orchards using terrestrial LiDAR point clouds. Furthermore, the maximum height of each olive was calculated by subtracting the highest height value of the pixels that composed the olive crown to the DTM. Then, the rest of the geometric features (width, length, and projected area) were automatically calculated for every crown tree object delimited in a previous step (*Tree crown delineation*) of the process. Finally, the geometric features of each olive, as well the identification and location, were automatically exported as vector (e.g., shapefile format) and table (e.g., Excel or ASCII format) files.

The algorithm was fully automatic and common for both planting patterns and training systems, with only one exception in the *Tree crown delineation* phase for the super high density continuous hedgerow (hedgerow trial) on the second date. In this training system, the tree exhibited adjacent canopies, starting the formation of a continuous canopy, i.e., a hedgerow trial, making it difficult to isolate the individual crown (**Figure 1C**). Thus, to solve this limitation, the location of every tree exported on the first date was used to identify each olive tree on the second date, so any square classified as “Tree” with its center at a distance of 1 m from the center of a tree was considered as part of that same tree. This 1 m-value was set taking into account the distance between trees. If no UAV image was available prior to the interception of the crowns, this issue could be solved by employing the planting pattern (distance between trees) or a grid with the position (x,y coordinates) of each tree.

Segmentation and slicing tasks are difficult, time consuming, and mostly performed by a human operator (Woo et al., 2002), thus the automation of these process in an OBIA algorithm enables objectivity and makes the olive characterization process time-efficient, reliable, and more accurate, removing errors from a subjective manual process.

### Validation

#### DTM Generation

The point cloud-based DTM created for each training system and date was compared to the official DTMs extracted from the IECA (Andalusian Institute for Statistics and Cartography, Spain), a public body that guarantees the organization, coordination, rationality, and efficiency of cartographic production in Andalusia (IECA, 2018). This official information is generally updated every 10–15 years and does not have enough high resolution in all areas of the region.

**Figure 5:**
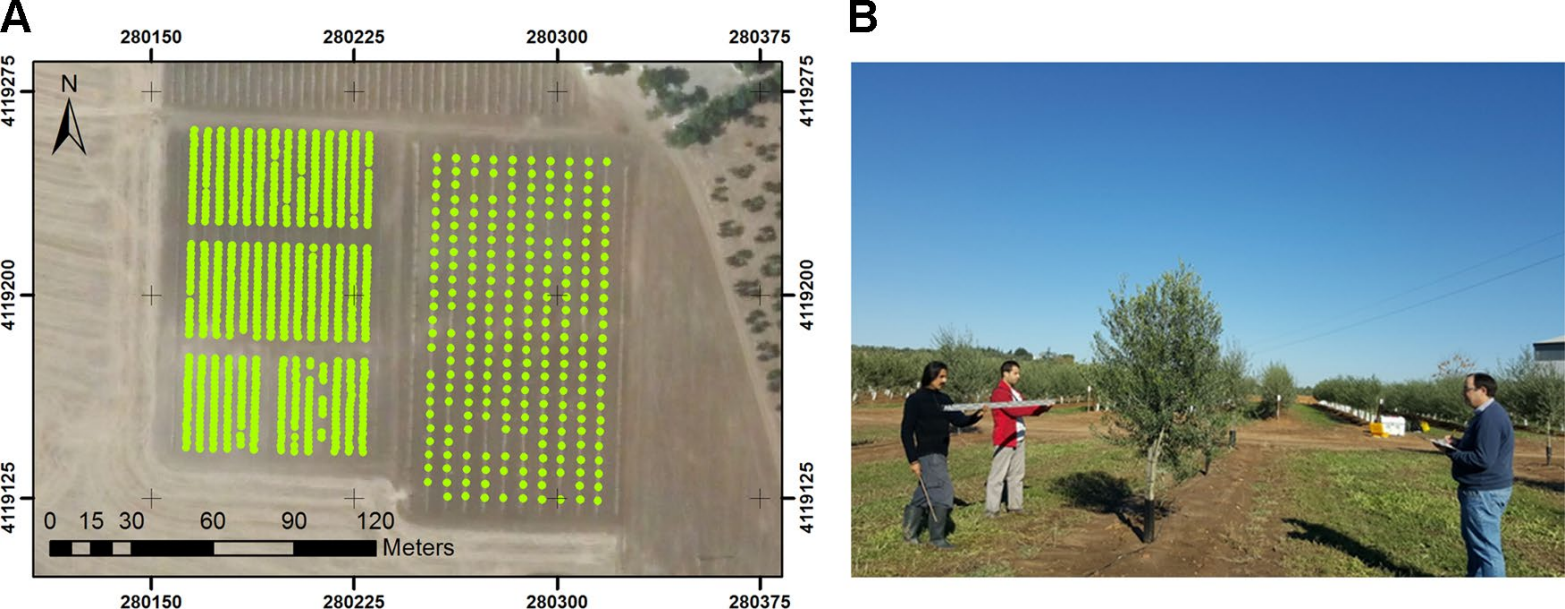
Experimental set for validation: **(A)** point grid for DSM validation in both the hedgerow and the intensive trial; **(B)** manual measurements of olive crown width. The individuals in this manuscript have given written informed consent to publish these case details.

The validation of the DTM was carried out on the basis of a 20 m grid over the studied fields by using ArcGIS 10.0 (ESRI, Redlands, CA, USA), resulting in 28 and 24 validation data points for the intensive and hedgerow training systems, respectively (**Figure 5A**). The distribution and quantity of the validation points made it possible to analyze the height variability in these field conditions. Then, the official IECA-DTM-based heights were compared to those estimated by the OBIA algorithm, and the coefficient of determination (R^2^) derived from a linear regression model was calculated using JMP software (SAS, Cary, NC, USA).

#### Olive Tree Identification and Geometric Traits Validation

Individual olive trees were visually identified in the mosaicked and compared to the image classification process outputs, i.e. the individual tree point cloud, and the coincidence was measured by calculating the counting accuracy (Eq. 1)

(1) $$Counting\;accuracy=\;\frac{OBIA\;identified\;olive\;trees\;}{Visual\;observed\;olive\;trees}\times 100$$

For tree geometric features validation, manually ground-based measurements of trees were taken in each field trial and date coinciding with the image acquisition (**Figure 5B**). Three geometric traits, namely tree height, crown area, and volume, were evaluated by comparing the OBIA estimated value and the on-ground observed values (true data). In the case of intensive trials, all traits were measured in each individual tree (244 trees); in the case of hedgerow trials, the tree height was also surveyed in all individuals (806 trees), and due to time and labor limitations, the canopy features were measured at 4 individual trees per elementary plot (164 trees). The validation trees were identified in the field and located their position in the mosaicked images.

The height of the tree, as measured up to the apex of the top of the tree, was taken with a telescopic ruler. In addition, the height and crown diameters (maximum projected horizontal width and its perpendicular) were acquired using a tape, and the crown area and volume were estimated assuming a circle (Eq. 1) and a cone-shaped (Eq. 2) form, respectively, applying validated methods (Eq. 2 and Eq. 3) for olive tree geometric measurements (Pastor, 2005), as follows:

(2) $$Field\;crown\;area=\pi {\left(\frac{D_{1}+D_{2}}{4}\right)}^{2}$$

where D_1_ is the is the widest length of the plant canopy through its center, and D_2_ is the canopy width perpendicular to D_1_.

(3) $$Field\;crown\;volume=\frac{Field\;crown\;area\times Measured\;canopy\;height}{3}$$

Then, the on-ground measures were compared to the OBIA-estimated values in order to assess the efficacy of the OBIA algorithm to estimate the olive traits of the very young plants. The coefficient of determination (R^2^) derived from a linear regression model was calculated using JMP software (SAS, Cary, NC, USA). The coefficient of determination (R^2^) is the proportion of the variance in the dependent variable that is predictable from the independent variable (Mendenhall et al., 2009), whereas the root mean square error (RMSE) is the standard deviation of the residuals, i.e. prediction errors (Barnston 1992). Additionally, the bias statistic was also calculated for the height comparison (Eq. 4), which measures the difference between the expected value of the estimator and the actual value of the parameter being estimated and evaluates its tendency to overestimate or underestimate that parameter (Lehmann 1951).

(4) $$Bias=\frac{y_{m}-x_{m}}{x_{m}}\times 100\%$$

where x_m_ is the mean height value of all field-measured trees, and y_m_ represents the mean detected OBIA height.

## Results

### Point Cloud and DTM Generation

**Figure 6:**
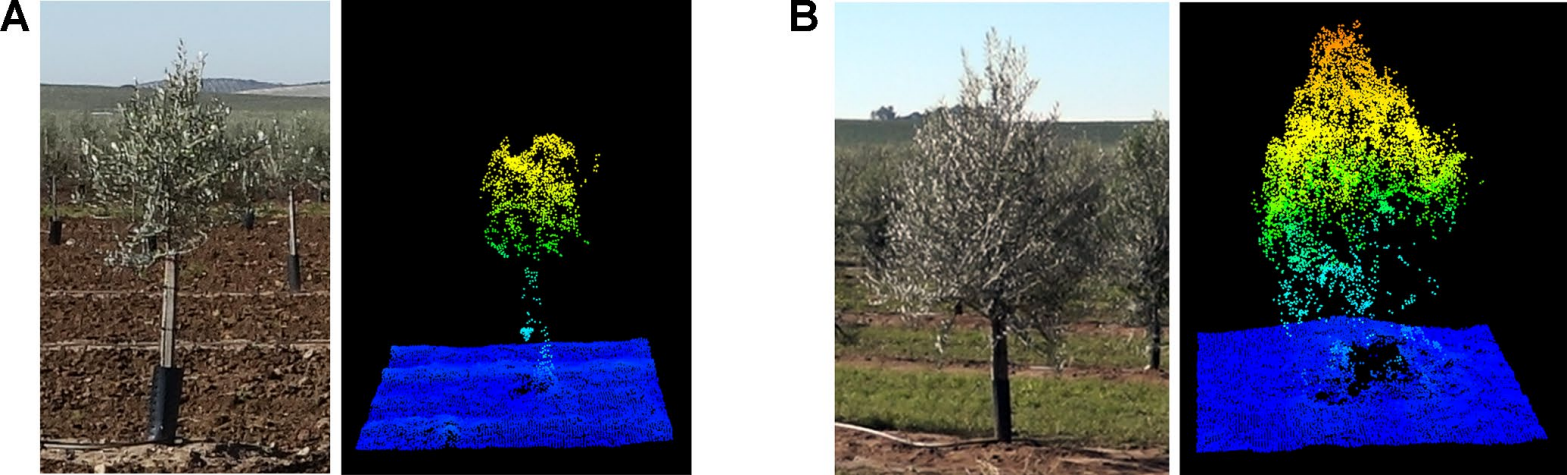
Field photograph and tree point cloud for an olive tree composing the intensive trial in the consecutive years of data collection: **(A)** January 2017—15 months after planting; **(B)** January 2018—27 months after planting.

High density point clouds were generated due to the large image overlap, based on the flight configuration, and the high spatial resolution of the UAV-imagery (**Figure 6**). The number of points in the cloud ranged from 4,136 points/m^2^ in the intensive trial in 2017 to 4,782 points/m^2^ in the hedgerow orchard in 2018 (**Table 1**). No major differences in point density were found between the training systems. However, the number of points was greater on the second flight date due to the larger size of the trees at the second flight date, i.e., 27 months after planting, as the ground point density remained constant. This greater number of points suggests that a higher accuracy in geometric features estimation could be reached, as there is a strong underlying control of the 3D reconstruction quality based on point cloud density (Dandois et al., 2015).

**Table 1:** Point density for each flight date and training systems.

Flight dates	Training systems	Point density (points/m^2^)
First year after planting	Intensive	4,136
January 2017	Hedgerow	4,152
Second year after planting	Intensive	4,441
January 2018	Hedgerow	4,782

As for the DTM, the algorithm generated it automatically and accurately from the point clouds achieving very high correlation with the official IECA-DTM for both intensive (R^2^ = 0.90) and hedgerow trials (R^2^ = 0.95), independently of the year and tree age. These results proved the suitability of the UAV-flight configuration to create appropriate point clouds as well as the performance of the OBIA algorithm for proper DTM generation. Some of the validation points for the hedgerow trial were dismissed in the comparison with the IECA-DTM as an anomalous area was found in this official DTM, making these validation points unusable.

### Olive Tree Detection

The OBIA algorithm successfully identified the olive trees, obtaining accuracy values higher than 93% in all of the studied cases (**Table 2**), with independence of the training system and olive age, which demonstrated the OBIA-algorithm’s robustness for tree detection at these early stages of growth. Furthermore, higher accuracy values were achieved in the later year of the study, i.e. 27 months after olive tree planting, reaching maximum precision or very close values, with results of 100% and 98.8% for intensive and hedgerow, respectively. This fact points out that, although it was possible to accurately create the tree point cloud and detect the olive trees at any of the studied olive ages, olive tree characterization could be affected by the plant age at these early stages.

**Table 2:** Accuracy attained by the OBIA algorithm in the olive tree detection.

Months after planting	Training systems	Field trees	OBIA detected trees	Accuracy (%)
15	Hedgerow	806	764	94.8
	Intensive	244	228	93.4
27	Hedgerow	804	794	98.8
	Intensive	243	243	100

### Olive Tree Characterization

#### Height of Olive Tree

A summary of the field height measurements and those estimated by OBIA for the matched trees in both studied dates and trials at field level is shown in **Table 3**. Height data for both training systems were analyzed by performing an analysis of variance (ANOVA) at the 0.05 level of significance by a Tukey Honestly Significant Difference (HSD) range test using JMP software (JMP 12, SAS Institute Inc., Campus Drive, Cary, NC, USA 27513) (**Table 3**). Significant differences in height data between hedgerow and intensive systems were observed in all comparisons, with the exception of the OBIA estimated outputs at the first date. This fact suggests that manual measurements were able to detect differences in height growth caused by the training system on both dates, where the algorithm could only do so on the second date.

**Table 3:** Summary of the field measured height and OBIA-estimated height for the matched trees at field scale.

	Months after planting	Training systems*	Minimum	Maximum	Range	Average	Standard deviation
Field data	15	Hedgerow	0.59	2.26	1.67	1.67a^§^	0.37
		Intensive	0.70	2.30	1.60	1.56b	0.28
	27	Hedgerow	1.15	3.25	2.10	2.51a	0.30
		Intensive	1.25	2.95	1.70	2.34b	0.31
OBIA data	15	Hedgerow	0.00	2.78	2.78	1.33a	0.55
		Intensive	0.34	2.28	1.95	1.30a	0.39
	27	Hedgerow	0.89	3.04	2.15	2.29a	0.32
		Intensive	1.25	3.09	1.84	2.22b	0.34

*764 and 228 trees for hedgerow and intensive trials, respectively in 2017; and 794 and 243 trees for hedgerow and intensive trials, respectively in 2018.§ For each column and months after planting mean values followed by different letter are statistically different at p = 0.05 (analysis of variance (ANOVA) by a Tukey Honestly Significant Difference (HSD) range test).

Analysis of variance of both measured and estimated height data between dates were also performed (data not shown) and significant differences were obtained in all of them, thus showing that both approaches (manual and estimated) detected the annual height growth. According to the field height measurements, height annual growth for intensive and hedgerow trials was 50.6% and 50.7%, respectively.

Based on the results shown in **Table 3**, the OBIA-estimated minimum values were lower than the field measurements, especially in 2017 due to the small size of some of the trees. The height estimates showed wider ranges of variation than the field measurements in all cases; although these differences were much smaller for the experiments in 2018. A similar trend was found for the average height, also obtaining greater agreement between the true and estimated measurements for the 2018 data. Thus, the height estimates were strongly influenced by the age of the olive plant at these growth stages, as stated above. It should be noted that the olive trees in the second year, i.e. 27 months after planting, were 3D reconstructed with a higher quality, as they showed values similar to those field measurements, suggesting that from this age, the estimation of this breeding trial at the individual tree level might be feasible.

**Figure 7:**
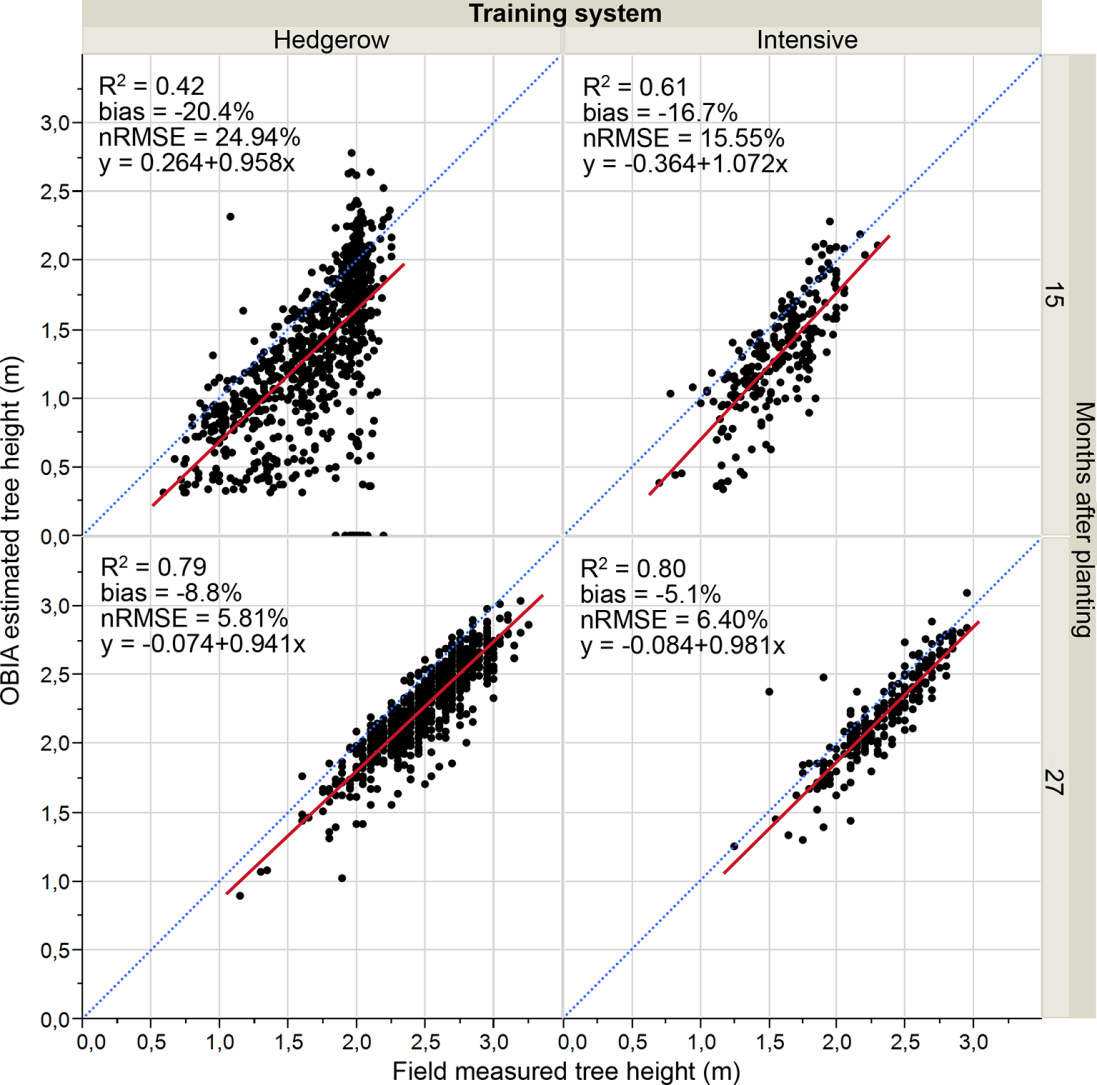
Point cloud-OBIA estimated height vs. measured olive height divided by training system and tree age. The relative root mean square error (nRMSE) and coefficient of determination (R^2^) derived from the regression fit are included for every scenario (p 0.0001). The red solid line is the fitted linear function and the blue dashed line represents the 1:1 line.

**Figure 7** shows the accuracy and graphical comparisons of the measured versus OBIA-estimated height at the individual tree level as affected by the pattern system and the olive tree age. As expected from the above results, correlations obtained for images taken in the first studied date (15 months after planting) were slightly lower than those reported for the second date (27 months after planting), i.e. after a growth cycle. At that first flight campaign, olive trees in an intensive pattern system achieved acceptable correlation values (R^2^ = 0.61), higher values than those reported by Díaz-Varela et al. (2015) using UAV imagery reconstruction based on DSM (R^2^ = 0.53) for height tree calculation of olive 5 years and 7 years after planting, i.e. with a larger size, which points out the feasibility of the our developed point-cloud based OBIA algorithm for the estimation of olive tree height in this growing system at the early age of 15 months after planting. No accurate results were obtained for hedgerow pattern at this first growth age.

Referring to the second flight campaign, low nRMSE (defined as the ratio of RMSE and the average value measured) values of 6.4 for intensive and 5.8 for hedgerow and R^2^ values around 0.80 were reported for both training systems. Similarly, De Castro et al. (2018a) reached a very high correlation (R^2^ = 0.78) in plant height estimation using UAV imagery and the OBIA approach in adult vineyards. Therefore, our results indicated that the OBIA algorithm accurately estimated the olive height at 27 months after planting, independent of the training system.

For both studied dates and growing systems, the comparison of the regression line with the 1:1 line and the negative bias indicated that the automatic OBIA algorithm underestimated the tree height parameter, especially on the first date that showed bias values higher than 16.7% for both patterns. The respective bias values for the second campaign images ranged from −5.1% to −8.8%, results comparable to those reached in the crown base height estimation of individual conifer trees in a forest scenario of 3.4% (Luo et al., 2018). Moreover, it should be pointed out that the underestimation was smaller for the intensive open vase orchard.

#### Olive Crown Parameters

**Figure 8:**
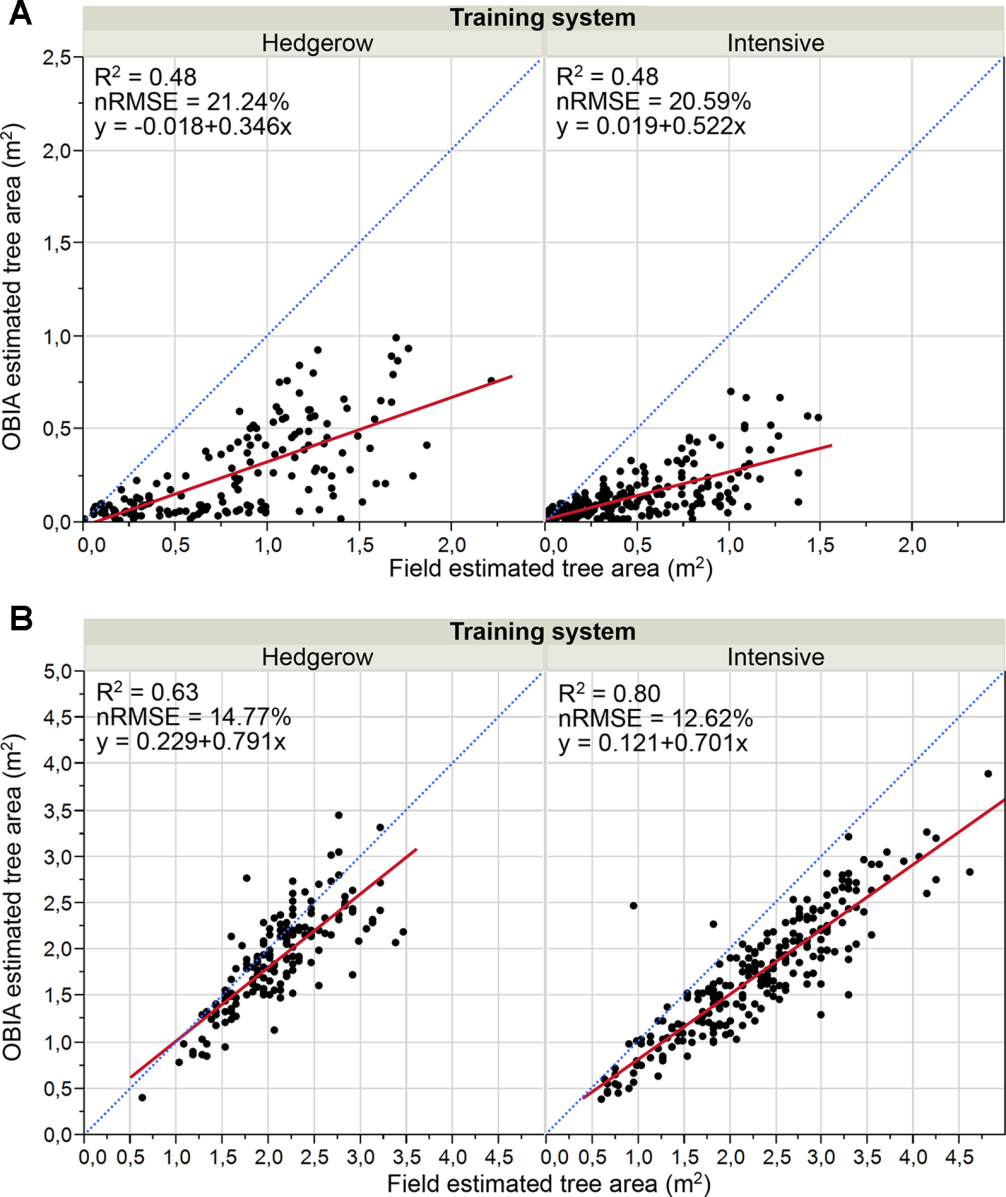
Graphical comparisons of Point cloud-OBIA estimated and field estimated crown area by training system in: **(A)** January 2017, 15 months after olive plantation; **(B)** January 2018, 27 months after olive plantation. The normalized root mean square error (nRMSE) and coefficient of determination (R^2^) derived from the regression fit are included for every scenario (p 0.0001). nRMSE was computed as the percentage of the average of measured values of tree variables. The red solid line is the fitted linear function and the blue dashed line represents the 1:1 line.

**Figure 9:**
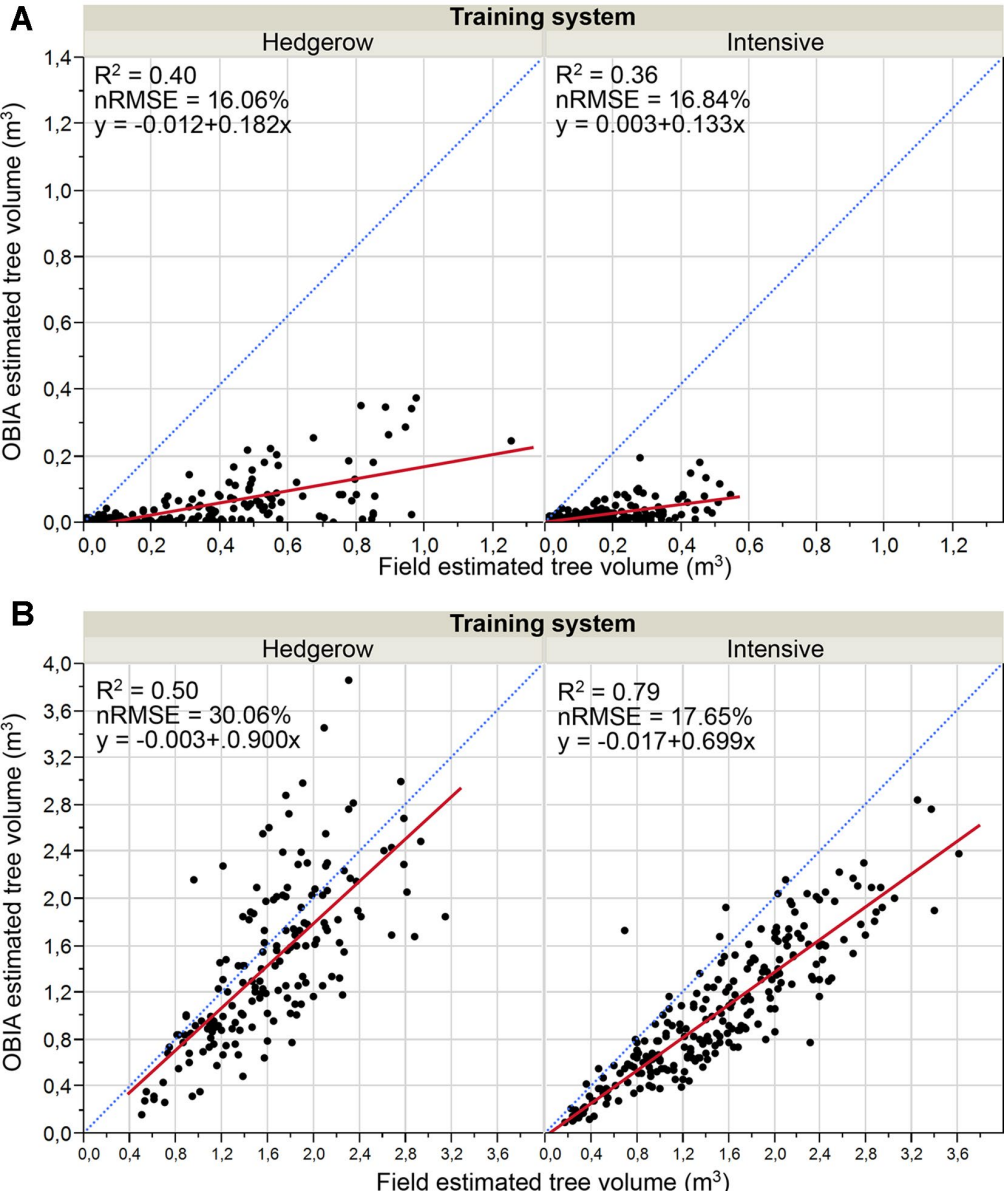
Graphic comparisons of Point cloud-OBIA estimated and field estimated crown volume by pattern system in: **(A)** January 2017, 15 months after olive plantation; **(B)** January 2018, 27 months after olive plantation. The normalized root mean square error (nRMSE) and coefficient of determination (R^2^) derived from the regression fit are included for every scenario (p 0.0001). nRMSE was computed as the percentage of the average of measured values of tree variables. The red solid line is the fitted linear function and the blue dashed line represents the 1:1 line.

Results of the validation work of the crown parameters, which consisted of comparing the OBIA estimated values to the calculated field data, are shown in **Figures 8** and **9** for areas and volumes, respectively. Much better correlations were achieved for both parameters in the 2018 data, i.e. the second flight campaign, thus following the same trend as the tree height. Similarly, the OBIA procedure also tended to a subtle underestimation of the crown parameters.

Analyzing the 2017 crown parameters results (**Figures 8A** and **9A**), both area and volume fits had low R^2^ with slight variations between the two crop systems, for example, R^2^ = 0.48 for the area parameter in both systems, and medium relative errors around 21%. In the case of the second campaign (**Figures 8B** and **9B**), the correlations were strengthened reaching higher determination coefficients, e.g. R^2^ values of 0.63 and 0.79 for the area comparison in the hedgerow system and volume in the intensive orchard, respectively. Minor errors were also reported for crown area comparison that ranged from 12.7% and 14.8%. And further, the points got much closer to the 1:1 line distribution, although they indicated a tendency to underestimate the results, as most of the points were below the 1:1 line. The better findings could be due to the bigger size of the tree at that date. In the analysis by training system in that latter campaign, a better fit of the OBIA estimated values to the manual truth data for canopy area and volume were achieved in the intensive orchard, which reported R^2^ of 0.79 and 0.80, respectively.

However, the determination coefficients for both crown parameters in the hedgerow orchards were slightly lower than those reported for the intensive one, e.g., R^2^ = 0.63 for area estimation (**Figure 8B**).

In summary, OBIA crown estimates of the olive trees in the first flight year were lower than those of the second year, i.e., 27 months after plantation, when the olive trees appear to reach a suitable size to be reconstructed using the point cloud-based OBIA algorithm developed. No clear differences in olive crown reconstruction were shown between both pattern systems using images at 15 months after olive plantation. Although the OBIA algorithm allowed the 3D reconstruction of the whole tree or hedgerow crown in the second year (**Figure 6**), better results were achieved in both the area and volume parameters for intensive orchards. In addition, the results showed a better fit for the canopy area than for the volume in all of the analyzed cases, reaching higher R^2^ values.

## Discussion

The objective of this research was to develop an UAV-based high-throughput system for olive breeding program applications, which consisted of UAV-flight configurations, in terms of flight altitude and image overlaps, and a novel, automatic, and accurate OBIA algorithm development. The system was evaluated in two experimental trials in the framework of the University of Sevilla table olive breeding program, with the aim to determine the earliest date for the suitable and precocious quantifying of architectural traits in large numbers of individuals under field conditions. Thus, two training systems were evaluated at two very early tree growth stages: 15 and 27 months after planting.

The flight configuration led to the generation of high density point clouds with around 4,500 points/m^2^ and the automatic and accurate DTM generation by means of the OBIA algorithm. In addition to the flight altitude and image overlap, which are defined in the flight configurations, the number of points are also strongly affected by the sensor spatial resolution (Dandois et al., 2015). In that sense, Torres-Sánchez et al. (2018b) obtained point clouds with half the density using the same flight configuration as in our experiment, but with a camera sensor size of 4,032 × 3,024 pixels, i.e. half the spatial resolution that the camera used in our study, which was quantified sufficient for the accurate detection of almond trees and the estimation of geometric characteristics. Therefore, both the sensor and flight configuration used in our research were considered suitable for DTM generation.

The accuracy of the DTM, directly affected by point cloud densities, is a critical issue for 3D tree characterization, as reported by Dandois et al. (2015) and Torres-Sánchez et al. (2018a) in woody crops, and the basis for the calculation of height-related trials (Dandois and Ellis, 2010), so the higher the quality in the DTM generation, the more precise the tree height estimation. In addition, the olive trees were also successfully identified by the OBIA algorithm from the DTMs, independent of the pattern system and olive age. The fact that no differences in tree detection and DTM generation were found between both training systems suggests that the growth patterns derived from each system were not significantly different at those tree ages for these specific goals, proving the robustness of this algorithm in those scenarios. Moreover, the accurate and automatic DTM created by the OBIA algorithm could be used not only as the first step in the procedure of quantifying breeding olive traits at early stages, but also as a valuable tool for generating accurate DTMs in agricultural studies, since the official DTMs extracted from the IECA are not always up-to-date, do not have enough spatial resolution in some areas of the region or accounts with faulty points, as previously reported. In addition, the automatic tree detection process, especially of small plants, could be a useful tool for some agricultural demanding tasks, for example, to count individual plants in nurseries due to their large fields and logistical considerations (De Castro et al., 2018c).

The OBIA algorithm was developed to generate agronomical traits considered key targets in olive phenotyping studies such as tree height, area, and volume of the crown, so that breeders can use those architectural traits to select the best genotypes according to desired objectives. For the olive tree height, the estimates were affected by tree size, and directly related to tree age, achieving much better accuracies for bigger olives, i.e., 27 months after planting. At that tree age, the tree height trait acquisition is feasible regardless of the evaluated training systems. However, only trees growing in the intensive system could be moderately reconstructed in the case of olive plants 15 months after planting, which may be because this system encourages free growth without any dominance directed from the height of the last tie (**Figure 1A**), making the trees reach less height, but in a uniform way in all the points of the crown (Innovagri, 2018). On the other hand, the hedgerow pattern employs a central axis formation system that promotes the growth of the terminal bud that acts as a guide, as opposed to the lateral shoots (**Figure 1B**), thus prioritizing height growth (Innovagri, 2018). These differences in growth due to training system become more accentuated in the early years. In fact, the growth pattern had a significant influence on the underestimation of the height trial, which was especially marked for the hedgerow pattern in 2017 as the generated point cloud did not detect the narrow apexes in the top of the olive trees due to this structure of the olive trees, which in contrast, were considered in the on-ground validation measurements. These findings are in agreement with those of Díaz-Varela et al. (2015) in an olive orchard of individual olive trees 5 and 7 years after plantation, and Peña et al. (2018) in a 1-year-old poplar plantation, in which the undervalues were assigned to the rough reconstruction of the final apex. Similarly, Kattenborn et al. (2014) found a general underestimation in the height trait of palm trees using UAV-based photogrammetric point clouds and stated that extend height deviations are indispensable, making difficult the sub-decimeter accuracy, which might be attributed to uncertainties in the reference data acquisition.

Similar considerations about automatic estimations were found for olive crown parameters: underestimation of the OBIA values; and much better correlations in the second studied date due to the greater crown size by the growth of the trees during the 12 months after the first campaign (**Figure 1** and **Table 2**), and for intensive orchards because of the more favorable growth pattern for measurements, as stated above. The underestimation of tree area and volume is common in automatic process, since the tree canopies are manually estimated by applying a conventional geometric equation that considers tree crown projection as circle forms and the tree crowns as cone-shaped forms while the actual trees have a more complex internal structure, with branches and void space within, which is captured by the algorithms using point clouds (Underwood et al., 2016). Thus, the assumption of a geometric shape for the crown, the complexity of taking on-ground tree measurements and the operator expertise may compromise the validity of field data (Torres-Sánchez et al., 2015; Sola-Guirado et al., 2017). These assumptions produce inexact on-ground estimations, while 3-D architecture derived from the point cloud-based OBIA algorithm reconstructed the irregular shape of the tree crown, achieving better estimations of the olive trials than those estimated from the on-ground measurements (Torres-Sánchez et al., 2015). In any case, similar trends and magnitudes between OBIA-estimated and field data were found, for example, the trees identified as bigger on the ground were also quantified as a larger area by the OBIA algorithm in 2018 (**Figure 8B**), and *vice versa*. This fact points out the suitability of the OBIA-based measurements for phenotyping trials, as it improves the traditionally considered errors of field estimates.

In olives 15 months after planting, neither the area nor the volume could be accurately estimated, showing that the tree point cloud was not dense enough to reconstruct the crown architecture at that growth stage (**Figure 6**). This matter could be solved by modifying the flight configuration either by reducing the flight altitude or using a higher resolution sensor so that the number of points are increased. In addition, this solution could resolve the underestimation of the traits from the OBIA algorithm, since a higher point density might lead to a better detection of tree apexes and part of the lateral branching, which caused the underestimation of crown parameters. However, the flight altitude has also strong implications in the flight duration, area covered by each image, time-consumption, image processing, spectral resolution, and cost (De Castro et al., 2015a). In this sense, flights at low altitude would increase the spatial resolution, i.e., more dense point cloud as well as the time and cost of the process (Gómez-Candón et al., 2014). Thus, an optimal combination of image overlap, sensor, and flight altitude is essential to optimize fieldwork for breeding applications in large-scale plant phenotyping studies. Therefore, a balance must be sought between the cost of refining the flight configuration and the earlier date to obtain the agronomic trait, i.e., the age of the plant, according to the desired target. Alternatively, the inclusion of oblique images in the analysis has shown potential to improve the DTM (James and Robson, 2014), although they have been mainly used for building damage assessment (Dong and Shan, 2013; Vetrivel et al., 2015) or quarry topography reconstruction (Rossi et al., 2017). Much less information exists on the use of oblique images for vegetation reconstruction that has been limited to forest trees after leaf fall (Fritz et al., 2013). Thus, a combination of nadir and oblique images could be tested in further research to check if this approach can improve the crown architecture reconstruction in agricultural vegetation. Apart from that, the underestimation issue could be solved applying an estimation corrector related to the tree characterization, age, and pattern system.

A higher level of agreement was reached in the second year, i.e. 27 months after planting, on the estimates of crown parameters, reaching a very high correlation and minor errors in both training system, and a slightly lower determination coefficient for volume in the hedgerow orchards, which could be attributed to inexact on-ground estimations, as stated above. Using a similar approach, i.e. UAV imagery and OBIA technology, Torres-Sánchez et al. (2015) estimated crown parameters with successful results both in single-tree and in hedgerow plantations, reporting R^2^ values of 0.94 and 0.65 for area and volume estimations, respectively, which proved that this technological combination is very suitable to obtain automatic and accurate agronomic traits. However, those experiments were carried out in adult trees, where actual crown volume ranged from 16 to 40 m^3^, making it a less complex scenario than that of olive trees shortly after planting. Comparatively, using that combination, weaker correlations (R^2^ = 0.58 and nRMSE = 18.83% for individual trees and R^2^ = 0.22 and nRMSE = 12.96% for hedgerow systems) were reached in crown diameter estimation when younger trees were analyzed (Díaz-Varela et al., 2015), which denotes an inverse relationship between both variables. Therefore, the accuracies obtained in this paper are considered highly satisfactory, since the experiments were carried out in the challenging initial growth stage of young olive trees.

In addition to its accuracy, this OBIA procedure was fully automatic, without any user intervention, making the quantification of the breeding trials time-efficient, reliable, and more accurate, removing errors from a manual intervention above explained (Jiménez-Brenes et al., 2017; De Castro et al., 2018b). In a previous research, Fernández et al. (2016) attempted to automatically detect olive using UAV-based point clouds, however, user intervention for manual point selection was required due to the difficulties they found in automatic identification, which led to a semi-automatic process that consumes time and resources, and could include a subjective element (De Castro et al., 2018b). Moreover, no field validation was performed by Fernández et al. (2016), so the use of a UAV-based point clouds methodology remained non-validated for olive trees. In this context, some authors have detected olive trees using UAV reporting classification accuracies over 90% (Torres-Sánchez et al., 2015; Jiménez-Brenes et al., 2017), although those studies were conducted under a DSM-based OBIA approach in adult olive trees. Therefore, our results are considered very successful as the automatic tree detection was carried out in very young olive orchards. Moreover, the time involved in the entire process took less than 5 h for the intensive orchard including 244 olive trees, which consisted of a 5 min flight; the point cloud generation, which took about 4 h; and running the algorithm, which was around 30 min. Thus, by using UAV-images in combination with the point-cloud based OBIA algorithm, an accurate DTM, number, and coordinates of each tree and their agronomic trials (height, area, volume) could be provided in the same day as UAV flights to breeders and farmers requesting plant architecture traits.

Rapid methods for identification and assessment of plant traits are considered a major challenge for crop research in the 21st century (White et al., 2012). The high-throughput system developed in this research can provide breeders demanded architectural traits as rapid as less than 5 h after flights. Moreover, this high-throughput system is able to 3D reconstruct olive trees around 1 year after plantation and calculates breeding traits as soon as 1 year or 2 years after plantation, depending on the trial and training system. Olive architectural traits are highly relevant in the evaluation of each breeding process stage: from the seedlings stage (De la Rosa et al., 2006; Rallo et al., 2008; Hammamia et al., 2012) to the advanced selections trials (Rallo et al., 2018), and are key to evaluate the adaptation of olive cultivars to new highly technified growing systems such as the super-high density hedgerows (Rosati et al., 2013; Rallo et al., 2014; Morales-Sillero et al., 2014). Furthermore, our UAV-based high-throughput system is cost and time optimized for large-scale plant phenotyping studies, so that the rapid, accurate, and timely outputs of this system could supply crucial information for the rapid selection of genotypes addressing, e.g., lower input demand, improved olive quality, the capacity to face threats such as *Xylella fastidiosa* or *Verticillium dahliae*, and climate change, among others (El Riachy et al., 2012; Fiorani and Schurr, 2013; Rallo et al., 2016).

Besides the breeding applications, this accurate and rapid obtainable information of plant traits and tree position in large fields could be useful to design precision agriculture strategies at orchard scale, such as fertilization, irrigation, designing of pruning tasks (Escolà et al., 2017; Peña et al., 2018; De Castro et al., 2018a), as well as site-specific canopy treatments at variable rate application adapted to the necessities and size of trees, which could result in savings herbicide of up to 70% (Solanelles et al., 2006). Nursery management could be also benefited from this technological system (De Castro et al., 2018c). Additionally, as the height tree and crown architecture estimation has been assessed in several training systems and growth stages, this technology could be used to evaluate the tree adaptation to different environmental conditions and/or growing systems (Díaz-Varela et al., 2015). In addition, the canopy monitoring throughout the growing cycle, together with the spectral information also provided in this approach, could open new opportunities for early identification of biotic and abiotic stresses, as visible- and near infrared range have been proved useful to detect early changes in plant physiology (De Castro et al., 2015b). Finally, the mapping of agronomical traits would help to address the goal of developing prediction models that connect olive growth traits to yield (Sola-Guirado et al., 2017).

This UAV-based high-throughput system has been designed by using UAV, GPS, and Agisoft PhotoScan Professional Edition Photoscan and eCognition Developer software for taking images, georeferencing the ground control points, generation of 3D point cloud, and identifying and characterizing young olive trees, respectively. The developed OBIA algorithm is self-adaptive to different crop-field conditions, as row orientation, row and tree spacing, field slope, or olive tree dimensions. Moreover, as the voxel methodology is used to calculate the volume, LiDAR point cloud could also be as input, though these systems are slower than UAV technology (De Castro et al., 2018a). Although none of the software used in this research are open access, these were selected due to their versatility to develop the rule-set that could be transferred to some open source software available in the market.

Alternatively, terrestrial laser scanners have shown potential for 3D tree characterization (Underwood et al., 2016; Luo et al., 2018). In this context, Escolà et al. (2017) used a 2D light detection and ranging (LiDAR) on board an all-terrain vehicle estimating olive crown volume with R^2^ values ranging from 0.56 to 0.82, depending on the algorithms used. The experiment was carried out in adult orchards, i.e., larger canopy sizes, and used a travel speed of 4 km/h, which requires more time. Moreover, LiDAR exhibits some weaknesses such as no spectral information is acquired, it is often difficult for it to hit the exact tops of trees (Luo et al., 2018), and problems of aligning LiDAR scans from both sides of the tree are reported (Rosell et al., 2009). Additionally, phenotyping platforms with ground vehicles are very difficult to use for cross-regional work due to the lack of maneuverability (Yang et al., 2017). On the other hand, higher point cloud densities were produced, which could imply a better 3D reconstruction, although none optimal densities have been proposed so far for agriculture (Escolà et al., 2017). Therefore, a comparison between tractor-mounted sensors and OBIA-UAV technology must be carried out in further research (Escolà et al., 2017; De Castro et al., 2018b).

In summary, the high-throughput system developed in this work consisted of UAV imagery and a robust point cloud based OBIA algorithm and allows the automatic, rapid, and accurate creation of Digital Terrain Models (DTMs) and identification of olive tree at any training system and age, as well as the extraction of olive architectural traits in large scale fields at a very young stage, that is, around 2 years after planting. In addition, tree height can be estimated with acceptable accuracy in an intensive trial at the first date, i.e. 15 months after planting. The early and accurate estimation of these traits through this cost-efficient methodology may drastically reduce the crucial time of decision making for tree breeders, therefore discarding the unwanted genotypes early and improving the performance of the breeding process (Fiorani and Schurr, 2013). Therefore, the time and cost saving of OBIA-based trait estimation as well as the higher accuracy, certainly justifies the utility of this technology rather than geometric assumptions based on manual measurement. Furthermore, the methodology may not only be applied in phenotyping tasks in olive breeding programs, but it will also support the modernization and intensification of the olive sector through a better management of these orchards, involving a beneficial effect on the market price of olive as well as the economic development especially in rural areas (White et al., 2012).

## Author Contributions

AIdC, PR, MPS, and FL-G conceived and designed the experiments. PR and MS designed the field trials and performed the olive field experiments. LC, AM-S, and MRJ collected and processed the ground-based data. AIdC, JT-S, FMJ-B, and FL-G performed the UAV flight experiments. AIdC, JT-S, and FMJ-B analyzed the data. FL-G, PR, and MPS contributed to the interpretation of the results, and with equipment and analysis tools. AIdC wrote the paper. FL-G and PR collaborated in the discussion of the results and revised the manuscript. All authors have read and approved the manuscript.

## Funding

The breeding field trials in which the experiments were performed are funded by Interaceituna (Spanish Inter-professional Association for Table Olives) through the FIUS projects PR201402347 and PRJ201703174. This research was partly financed by the AGL2017-83325-C4-4-R (Spanish Ministry of Science, Innovation and Universities and AEI/EU-FEDER funds), and Intramural-CSIC 201940E074 Projects. Research of AC was supported by the Juan de la Cierva Program-Incorporación of the Spanish MINECO funds.

## Conflict of Interest

The authors declare that the research was conducted in the absence of any commercial or financial relationships that could be construed as a potential conflict of interest.

## Supplementary Material

Animations of several figures of the paper are available as supplementary materials (**Supplementary Video File**) in the article electronic version, published online. These might be used by the interested reader in order to have a dynamic representation of the various processing phases.
